# Ecosystem services changes between 2000 and 2015 in the Loess Plateau, China: A response to ecological restoration

**DOI:** 10.1371/journal.pone.0209483

**Published:** 2019-01-28

**Authors:** Dan Wu, Changxin Zou, Wei Cao, Tong Xiao, Guoli Gong

**Affiliations:** 1 Nanjing Institute of Environmental Sciences, Ministry of Environmental Protection, Nanjing, China; 2 Key Laboratory of Land Surface Pattern and Simulation, Institute of Geographic Sciences and Natural Resources Research, CAS, Beijing, China; 3 Satellite Environment Center, Ministry of Environmental Protection, Beijing, China; 4 Shanxi Academy of Environmental Planning, Taiyuan, China; Oak Ridge National Laboratory, UNITED STATES

## Abstract

The Loess Plateau of China is one of the most severe soil and water loss areas in the world. Since 1999, the Grain to Green Program (GTGP) has been implemented in the region. This study aimed to analyze spatial and temporal variations of ecosystem services from 2000 to 2015 to assess the effects of the GTGP, including carbon sequestration, water regulation, soil conservation and sand fixation. During the study period, the area of forest land and grassland significantly expanded, while the area of farmland decreased sharply. Ecosystem services showed an overall improvement with localized deterioration. Carbon sequestration, water regulation and soil conservation increased substantially. Sand fixation showed a decreasing trend mainly because of decreased wind speeds. There were synergies between carbon sequestration and water regulation, and tradeoffs between soil conservation and sand fixation. It was concluded that ecological projects have contributed significantly to the rehabilitation of the fragile ecosystems of this region. To make these projects more successful and sustainable, long-term management procedures are necessary to maintain and consolidate the improvements.

## Introduction

Ecosystem services are the benefits that people derive from nature, including both tangible products and intangible services [1], which can be divided into supporting services (soil formation, nutrient cycling), provisioning services (food, wood, water), regulation services (water regulation, climate regulation, land degradation) and cultural services (entertainment, consciousness, religion) [2]. These benefits contribute immensely to human welfare and play a vital role in supporting life on the planet [3]. However, because of the accelerated growth of industrialization, urbanization and agricultural modernization, in addition to severe population pressure and excessive demands on natural resources, 60% of worldwide ecosystem services have degraded, which poses direct threats to regional and global ecological security [2]. Natural ecosystems show development trends from structural damage to functional disorder [4]. As a result, ecological problems are exacerbated, such as shrinkage of wetlands, soil erosion, water pollution, loss of carbon sinks, decreasing biodiversity, and increasing sand storms, droughts and floods. The sustainable development of regional societies, economies and ecosystems has become an important focus for the scientific community. GIS and remote sensing technologies development and their application in ecological research has provided dynamic spatial support for the comprehensive assessment of multiple ecosystem services [5–6]. The main challenges for ecosystem services studies at present are trade-offs, scaling, accounting and assessment, modeling and scenario planning [7].

In China, widespread ecological degradation has constrained sustainable socioeconomic development in recent decades, particularly towards the end of the 20^th^ century [8]. For instance, degraded areas occupied 23% of China from 1981–2003, which provided for about 35% of the Chinese population. About 21% of degraded land was arable, 39% was forest, and 31% was grassland and scrub [9]. More than 90% of the natural grasslands in China were deteriorating at different levels, and the deterioration was accelerating at a rate of 2% annually in the available grassland regions [10]. At the same time, serious natural disasters suffered by China in 1997 and 1998 spurred new efforts to protect the fragile and fragmented environment [11]. Because of severe drought in 1997, lower reaches of the Yellow River remained dry for up to 267 days, jeopardizing water availability and agricultural development [12]. In 1998, massive floods along the Yangtze River and waterways claimed more than 3000 people’ lives and led to more than 12 billion dollars of property damage and production losses [13]. These problems affected the security of people's lives and properties. The degradation of these ecosystems reduced their capacity to support human activities and regional sustainable development. In the wake of natural disasters and ecological degradation, the Chinese government shifted its focus from economic progress to environmental protection. This included rehabilitation of damaged forest ecosystems, afforestation in degraded areas, and a ban on logging in natural forests [14]. Since then, several ecological restoration programs have been undertaken in China, especially the Six Key Forestry Programs [15]. Investments in the forestry sector since 2000 have exceeded the total investments over the period 1949–99 [16]. The Natural Forest Conservation Program (NFCP) and the Grain to Green Program (GTGP) are the biggest of the six programs and have ambitious goals, massive scales, huge payments and potentially enormous impacts [17]. Effectively evaluate the long-term ecological effects of these programs and incorporate ecosystem service changes into policy making and planning have become urgent problems for China [18].

The Chinese Loess Plateau is considered to be one of the most severe soil and water loss areas in the world [19], and has been largely degraded by deforestation and cultivation, combined with the effects of heavy rainfalls in summer, steep topography and highly erodible loess soil [20]. Soil erosion has become a major environmental threat in this region, and this has seriously depleted land resources, degraded the environment and directly affected local agricultural and industrial productivity. The GTGP ecological restoration program was initiated in 1999 to relieve soil erosion and enhance ecosystem services. Many areas of steep slope croplands have been converted to forested lands or grasslands. The implementation of GTGP has further enhanced vegetation restoration and ecological conservation. At the same time, the coupling and feedback between natural conditions and ecological effects have caused wide public concern in recent years.

This paper focuses on the land use and ecosystem services changes since implementation of the GTGP in the Loess Plateau through spatial analysis and model simulation. The objectives of this study during the period of 2000–2015 were to (1) quantitatively analyze land use changes; (2) quantitatively evaluate spatial and temporal changes in ecosystem services (carbon sequestration, water regulation, soil conservation and sand fixation); and (3) assess the effects of the GTGP implementation. Findings from this study could provide guidance for ecological protection policy-making and planning at regional scale in the future.

## Materials and methods

### Study area

**Fig 1:**
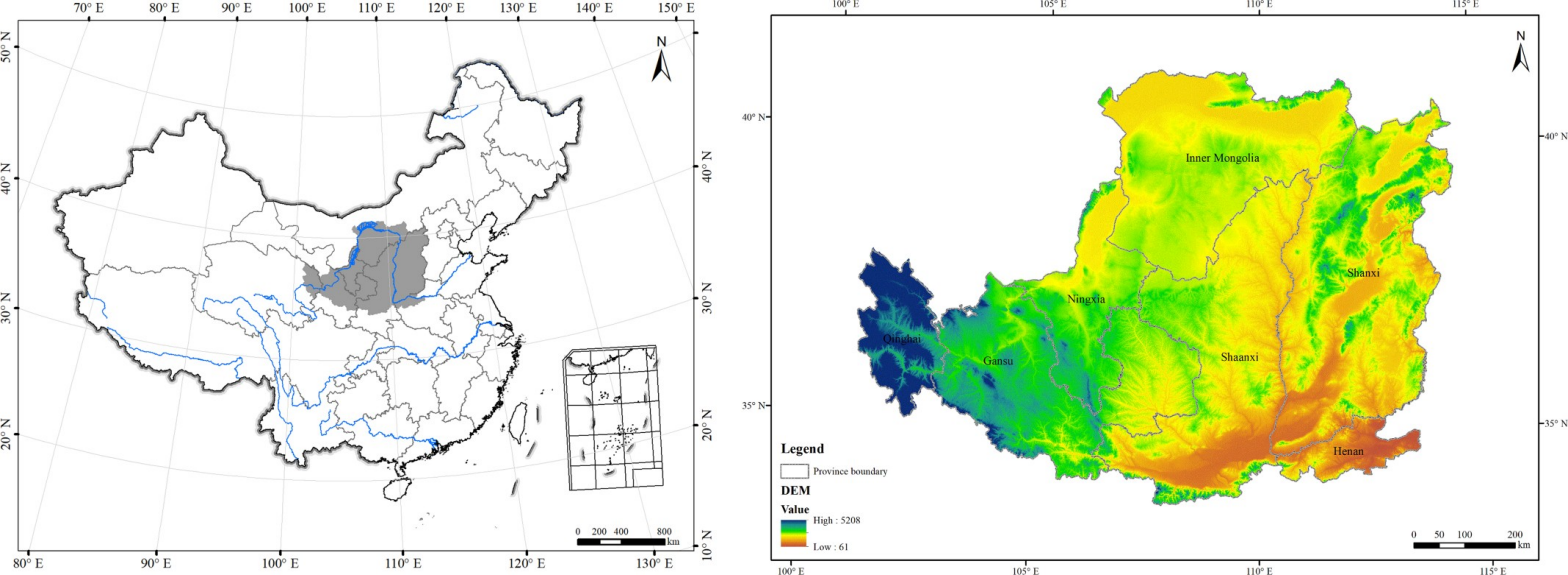
Location of the Loess Plateau, China.

The Loess Plateau region (33°43'–41°16'N, 100°54'–114°33'E) is located in the middle reaches of the Yellow River Basin, northwestern China (Fig 1), extending to the Qinling Mountains in the south, the Yinshan Mountains in the north, the Taihang Mountains in the east and the Riyue Mountains in the west. This region covers seven provinces, including Shanxi, Shaanxi, Henan, Ningxia, Gansu, Qinghai and Inner Mongolia. As the most concentrated and largest distribution of loess, it covers an area of about 625,000 km^2^, 6.50% of the territory in China. It has complex topography, including basins, sub-plateaus, hills and gullies, with elevation ranging from 60 to 5200 m. It experiences a continental monsoon climate. Annual temperature is about 6 to 14°C, and annual precipitation is 200 to 700 mm. Approximately 70% of annual precipitation occurs between June and September. The Plateau surface is covered by highly erodible loess layers and the soil types are mainly clayey loess and typical loess. Natural vegetation types vary from broad-leaved deciduous forest, to steppe and then to arid desert in the direction from southeast to northwest [21].

### Ecosystem services assessment

To quantify and map multiple ecosystem services in the Loess Plateau, four dominant ecosystem services (carbon sequestration, water regulation, soil conservation and sand fixation) were assessed based upon biophysical models and field observations. The carbon sequestration was calculated using Biome-BGC and Radiation Use Efficiency (RUE) model. The water regulation function was simulated by the precipitation storage method. Soil conservation and sand fixation were estimated according to the Revised Universal Soil Loss Equation (RUSLE) and Revised Wind Erosion Equation (RWEQ), respectively. For the models above, all parameters were localized to improve the accuracy of results.

### Carbon sequestration assessment

Annual net primary productivity (NPP) was calculated as the gross primary productivity (GPP) less the costs associated with annual maintenance and growth respiration [22]. The main equations are as follows:

NPP=GPP‑Livewood_MR‑Leaf_MR‑Froot_MR‑Leaf_GR‑Froot_GR‑Livewood_GR‑Deadwood_GR (1)

GPP=ε*APAR (2)

Where Livewood_MR, Leaf_MR, Froot_MR are maintenance respiration (gC) of live wood, leaves and fine roots, respectively. Leaf_GR, Froot_GR, Livewood_GR and Deadwood_GR are growth respiration (gC) of leaves, fine roots, live wood and deadwood, respectively. ε is the PAR conversion efficiency (gC/MJ). APAR is the absorbed photosynthetically active radiation (MJ/m^2^).

#### Water regulation assessment

The water regulation capacity of forest and grassland ecosystems was calculated by the precipitation storage method [23]. Formulae are calculated as:

Q=A∙J∙R (3)

$$J=J_{0}∙K$$ (4)

$$R=R_{0}‑R_{g}$$ (5)

where Q is water regulation capacity of ecosystems such as forest or grassland compared with bare land (m^3^); A is the ecosystem area (ha); J is annual output of flow rainfall (mm); J_0_ is annual precipitation (mm); K is the percentage of annual output of flow rainfall to precipitation amounts; R is the profit coefficient of ecosystems’ decreasing runoff; R_0_ and R_g_ are the rainfall-runoff rate of bare lands and ecosystems, respectively.

#### Soil conservation assessment

Soil conservation (A_c_) was defined as the potential soil loss in conditions of extreme degradation minus the soil loss with the current land use/land cover [24]. The equation is:

$$A_{c}=A_{p}‑A_{r}$$ (6)

where A_p_ is the soil loss (t/ha/yr) in conditions of extreme degradation without vegetation cover and A_r_ is the soil loss with the current land cover and management conditions.

Soil loss intensity was calculated using the Revised Universal Soil Loss Equation (RUSLE) [25]. The RUSLE equation is:

A=R×K×L×S×C×P (7)

where A is annual soil erosion module (t/ha/yr); R is rainfall erodibility factor (MJ·mm/(t/ha/yr)); K is soil erodibility factor (t·h/(MJ·mm)); L is slope length factor; S is slope factor; C is vegetation cover factor and P is erosion control practice factor. Factors C and P are dimensionless.

#### Sand fixation assessment

Sand fixation (S_c_) was defined as the potential wind erosion of bare soil conditions minus wind erosion with the current land use/land cover. The equation is:

$$S_{c}=SL_{p}‑SL_{r}$$ (8)

where SL_p_ is annual wind erosion (t/ha/yr) in conditions of bare soil and SL_r_ is annual wind erosion with current land cover.

Wind erosion intensity was calculated using the Revised Wind Erosion Equation (RWEQ) [26]. The RWEQ equation is:

$$\mathrm{SL}=Q_{max}\left[1-e^{{\left(x/s\right)}^{2}}\right]$$ (9)

$$Q_{max}=109.8\times \left(\mathrm{WF}\times \mathrm{EF}\times \mathrm{SCF}\times K^{\prime }\times C\right)$$ (10)

where SL is annual wind erosion module (t/ha/yr); Q_max_ is the maximum transport capacity (kg/m); x is field length (m); s is critical field length (m); WF is climate factor (kg/m); EF is soil erodibility factor; SCF is soil crust factor; K' is soil roughness factor; and C is vegetation cover factor.

#### Interactions among ecosystem services

Interactions between pairs of ecosystem service were estimated through correlation analysis [27], which was performed on each pair of services using SPSS statistical software. Correlations were analyzed using the Pearson parametric correlation test.

#### Vegetation coverage change

Vegetation cover (VC) was calculated through the Normalized Difference Vegetation Index (NDVI) dataset using the dimidiate pixel model method according to the following equation [28]:

$$\mathrm{VC}=\frac{NDVI-NDVI_{soil}}{NDVI_{veg}-NDVI_{soil}}$$ (11)

where NDVI is the value for each pixel; NDVI_veg_ and NDVI_soil_ is the NDVI values of pure vegetation and bare soil, respectively.

The VC change was divided into five categories according to change of slope: obvious degradation (slope≤−0.5%), slight degradation (−0.5<slope≤−0.2%), stable (−0.2<slope≤0.2%), slight restoration (0.2<slope≤0.5%), and obvious restoration (slope>0.5%).

#### Change trend analysis

The annual variation trends for ecosystem services and climatic factors were calculated by the slope equation [29], which can be calculated for a certain period and for each pixel in ArcGIS. It is expressed as:

$$\mathrm{Slope}=\frac{n\times \sum_{i=1}^{n}i\times X_{i}-\sum_{i=1}^{n}i\sum_{i=1}^{n}X_{i}}{n\times \sum_{i=1}^{n}i^{2}-{\left(\sum_{i=1}^{n}i\right)}^{2}}$$ (12)

where i is the number of years; n is the total number of years; and X_i_ is value of variable for each year i. Positive values indicate an overall increase or restoration trend, while negative values of the slope represent the occurrence of degradation and.

#### Data sources and processing

Land use datasets (30 m) for 2000 and 2015 were supplied by the Satellite Environment Center, Ministry of Environmental Protection. Meteorological data (daily rainfall, temperature, wind speed, relative humidity, and sunshine duration) from 2000 to 2015 were provided by the National Meteorological Information Center (http://data.cma.cn/) from 136 national meteorological stations; 72 were located in the Loess Plateau and the remaining 64 were adjacent to the Plateau. Soil type and texture data (1 km) were collected from the Resource and Environmental Science Data Center, Chinese Academy of Sciences (http://resdc.cn/). DEM Dataset (90 m) was derived from Geospatial Data Cloud (http://www.gscloud.cn/). The gridded yearly 1 km NPP (Net Primary Productivity) MOD17A3 products (2000–2015) and 16-day 1 km NDVI (Normalized Difference Vegetation Index) MOD13A products (2000–2015) were obtained from NASA EOS DATA (https://modis.gsfc.nasa.gov/data/). The rainfall-runoff rate for different vegetation types and P values were collected through a literature review. The meteorological station data were spatially interpolated into raster grids using the Anusplin method. Raster data with different resolutions were resampled into the same cell size of 1 km. All data (or layers) needed in the process of water regulation, soil conservation and sand fixation modeling are shown in S1 Fig, S2 Fig and S3 Fig.

## Results

### Land use changes

**Fig 2:**
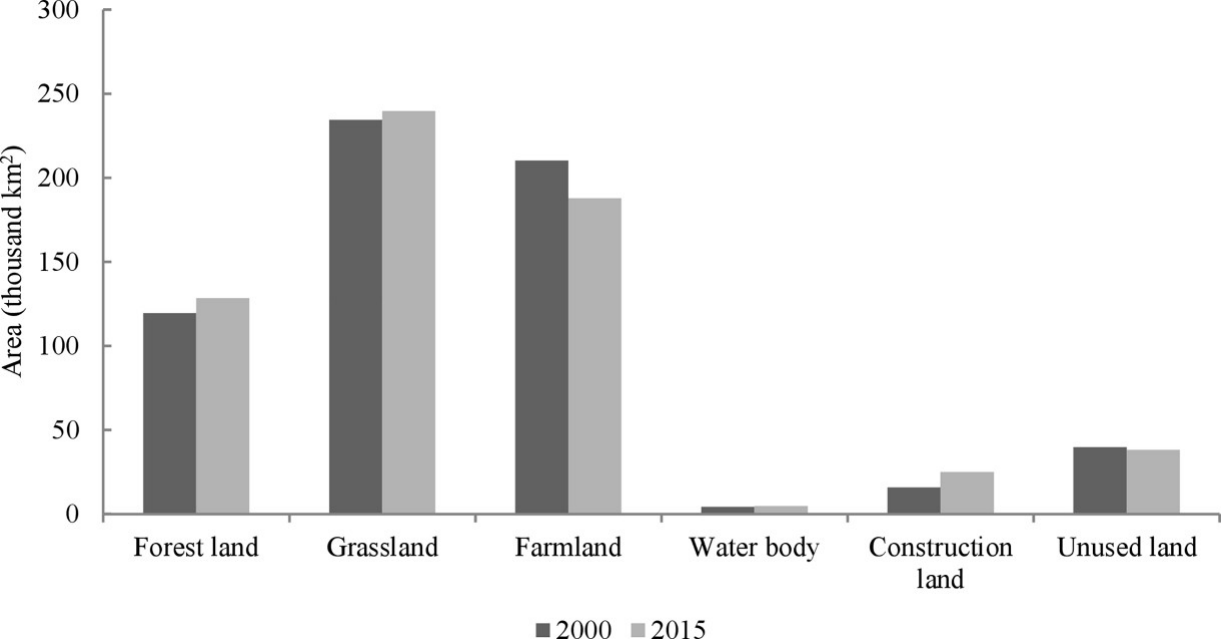
Areas of land use types in the Loess Plateau in 2000 and 2015.

**Fig 3:**
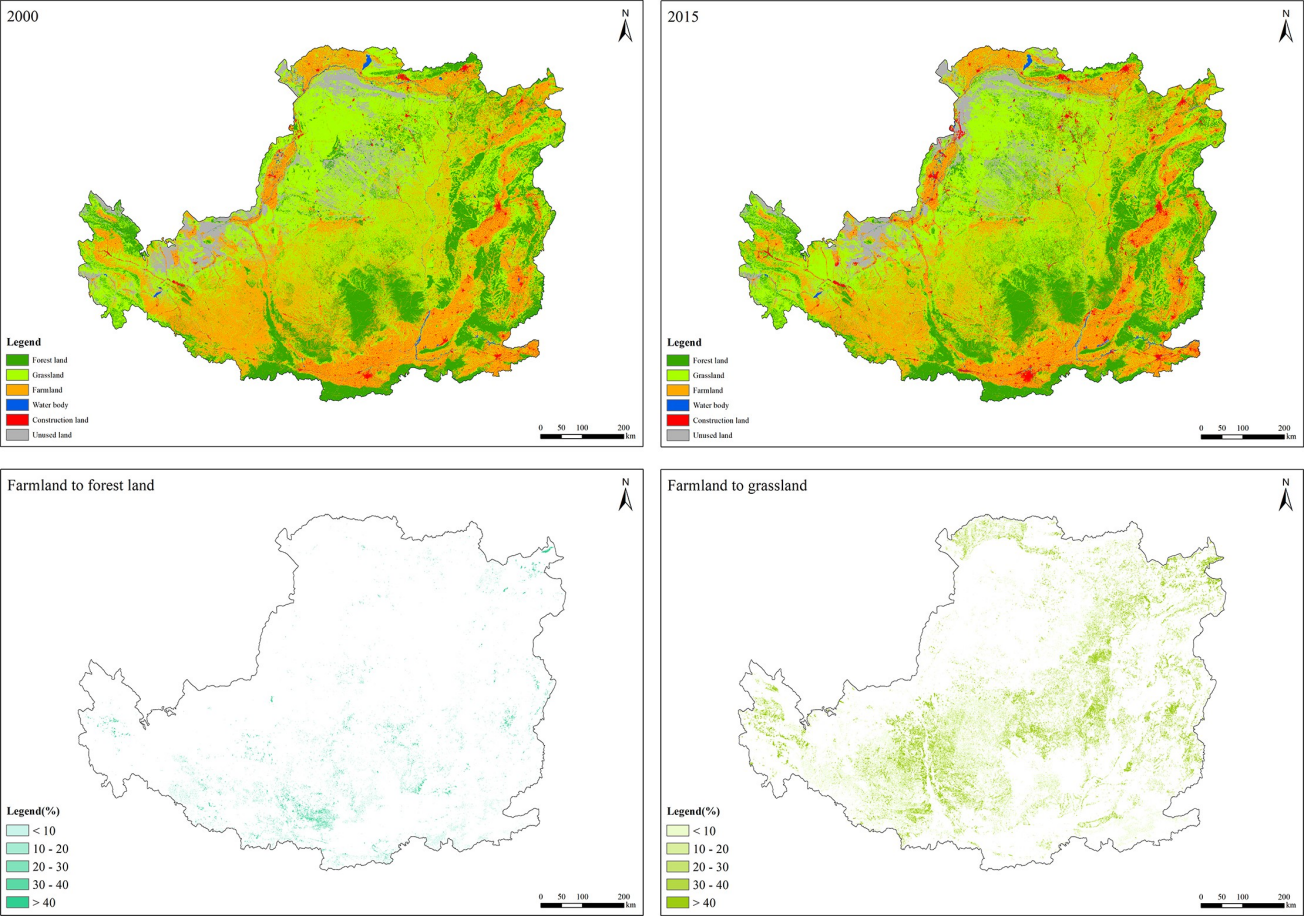
Distribution and main spatial changes of land use types in the Loess Plateau from 2000 to 2015.

The Loess Plateau was dominated by grasslands and farmlands prior to the GTGP implementation. Land use changes in the Loess Plateau between 2000 and 2015 were shown in Figs 2 and 3. The land use patterns changed remarkably during this period. Forest land, grassland and water body areas increased by 7.49%, 2.23% and 9.17%, respectively. Conversely, farmland and unused land areas decreased by 10.58% and 3.91%, respectively. Construction land area rose sharply and had the highest increasing rate (58.34%) because of the increasing encroachment of human activities. Forest land and grassland areas increased by 8954.37 km^2^ and 5235.38 km^2^, respectively, while farmland area decreased by 22238.34 km^2^ in the period from 2000–2015.

### Carbon sequestration changes

**Fig 4:**
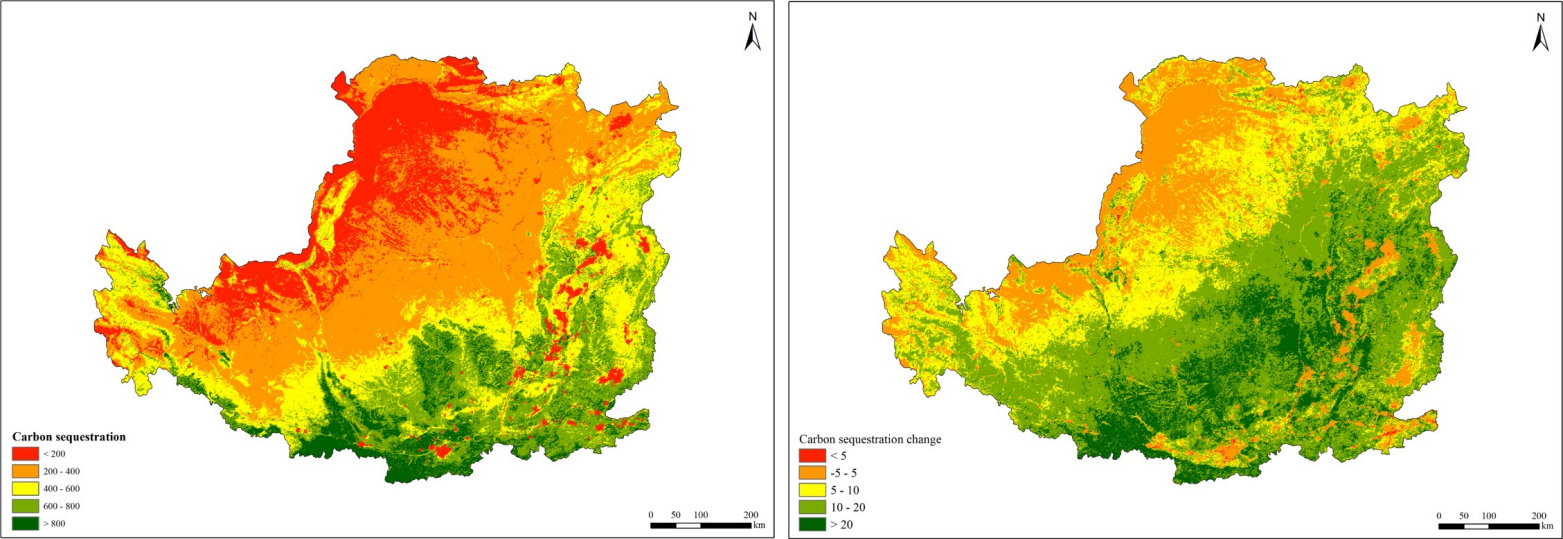
Spatial distribution of carbon sequestration in the Loess Plateau from 2000 to 2015.

The average carbon sequestration during 2000 and 2015 was 415.34 gC/m^2^/yr, and this increased annually by an average of 11.50 gC/m^2^, which suggested that ecological rehabilitation efforts have brought significant positive impacts on carbon sequestration. The carbon sequestration capacity was higher in the southeast of the Loess Plateau and lower in construction land and unused land (Fig 4). The carbon sequestration capacity of forest land, grassland and farmland ecosystems was 61.71, 32.79 and 45.48 gC/m^2^/yr, respectively. The spatial variation in carbon sequestration during the study period was evident from southeast to northwest. More than 90% of the Loess Plateau showed an increase in carbon sequestration during the study period.

### Water regulation changes

**Fig 5:**
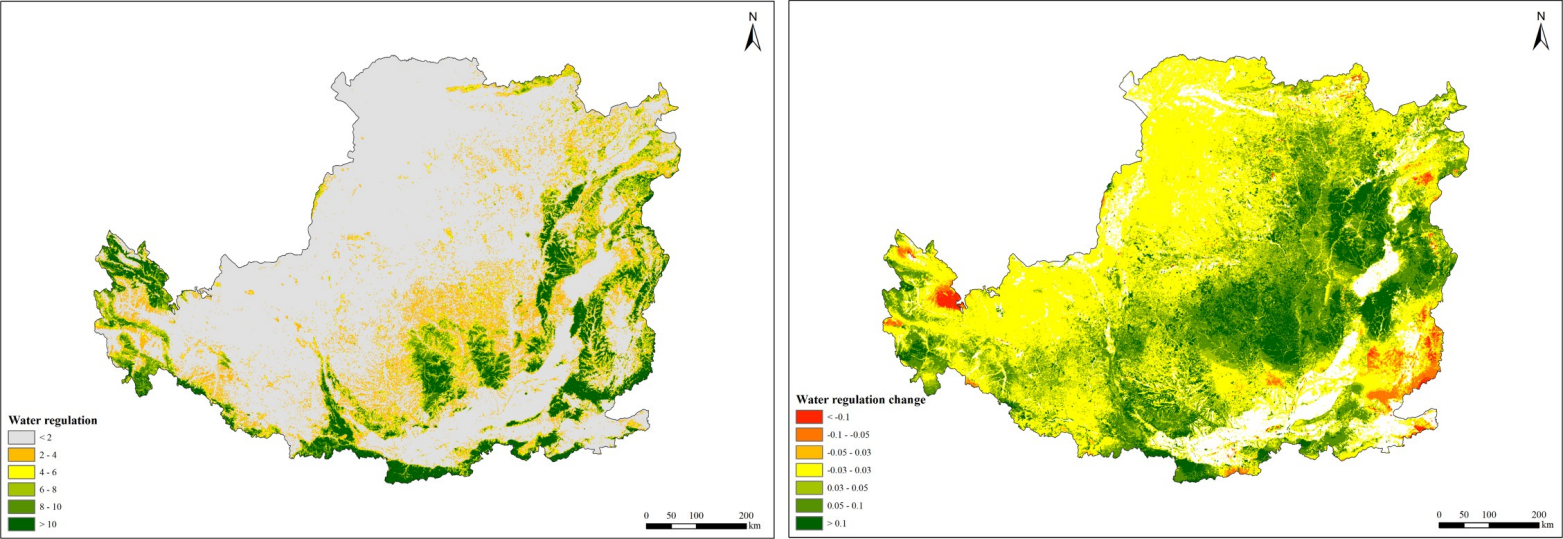
Spatial distribution of water regulation in the Loess Plateau from 2000 to 2015.

Water regulation capacity of forest and grassland ecosystems improved from 215.92 m^3^/ha/yr in 2000 to 241.09 m^3^/ha/yr in 2015. During this period, average annual water regulation amount was about 16.52 billion m^3^/yr, with an increasing trend of 0.21 billion m^3^/yr. Water regulation capacities of forest and grassland ecosystems were 160.86 and 103.86 m^3^/ha/yr, respectively. Water regulation capacity had obvious regional differences as it was mainly affected by the distribution patterns of natural ecosystems and precipitation. Water regulation capacity in the Taihang, Lvliang and Zhongtiao Mountains of Shanxi Province, the Qinling and Daba Mountains of Shaanxi Province and eastern Qinghai was higher than in other areas.Water regulation capacity had improved between 2000 and 2015 for most areas in the Loess Plateau (Fig 5).

### Soil conservation changes

**Fig 6:**
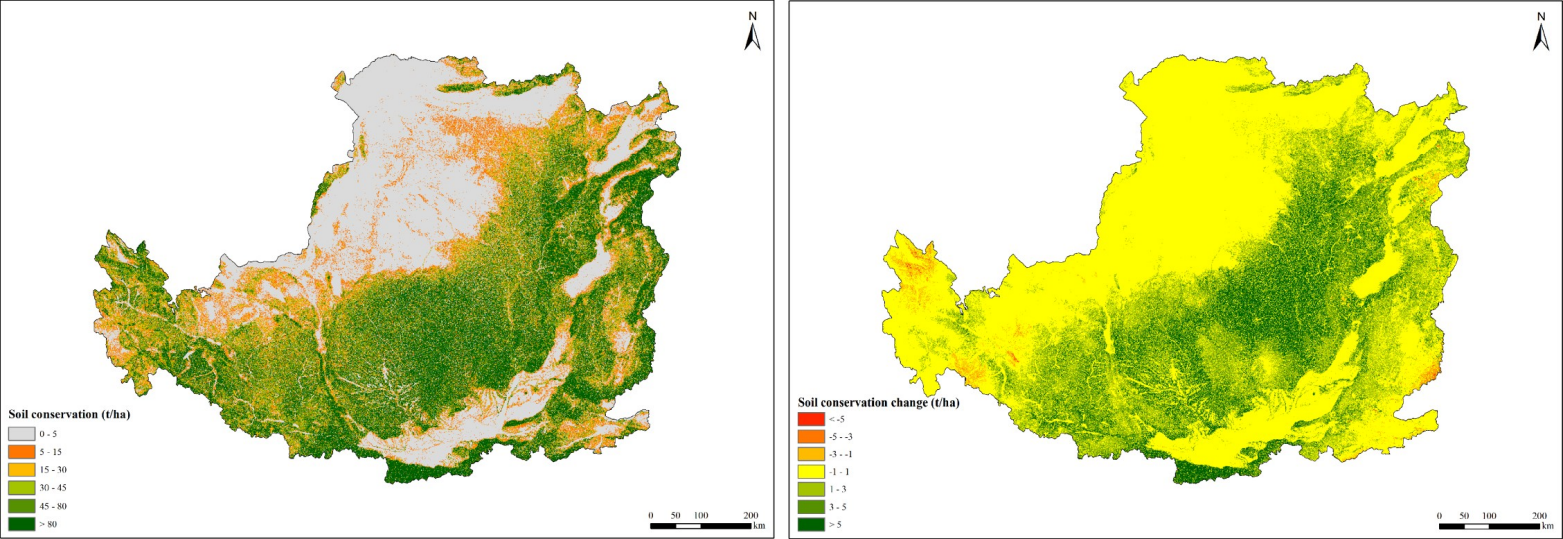
Spatial distribution of soil conservation in the Loess Plateau from 2000 to 2015.

Soil conservation in the Loess Plateau showed substantial improvement from 2000 because of vegetation restoration (Fig 6).Average annual amount of soil conservation was 2.89 billion t/yr and the per unit area was 46.33 t/ha/yr from 2000 to 2015. Although soil conservation capacity fluctuated year by year, it showed an overall positive trend of 1.54 t/ha/yr. The soil conservation capacity of different ecosystems showed great differences. The soil conservation capacity of farmland ecosystems was the lowest, with an average per unit area of 29.29 t/ha/yr during the study period. Grassland ecosystems were intermediate, with an average per unit area of 40.04 t/ha/yr, which was 1.37 times higher than that of the farmland ecosystems. Forest ecosystems had substantially higher soil retention capacity (101.46 t/ha/yr) than those of other ecosystems. The soil conservation capacity was low in Middle Shanxi and in the farming areas of Southern Qinling, Erdos Platea, Hetao Plain and Ningxia Plain. The spatial variation trend of soil conservation during the study period was improved in vast majority of areas (Fig 6).

### Sand fixation changes

**Fig 7:**
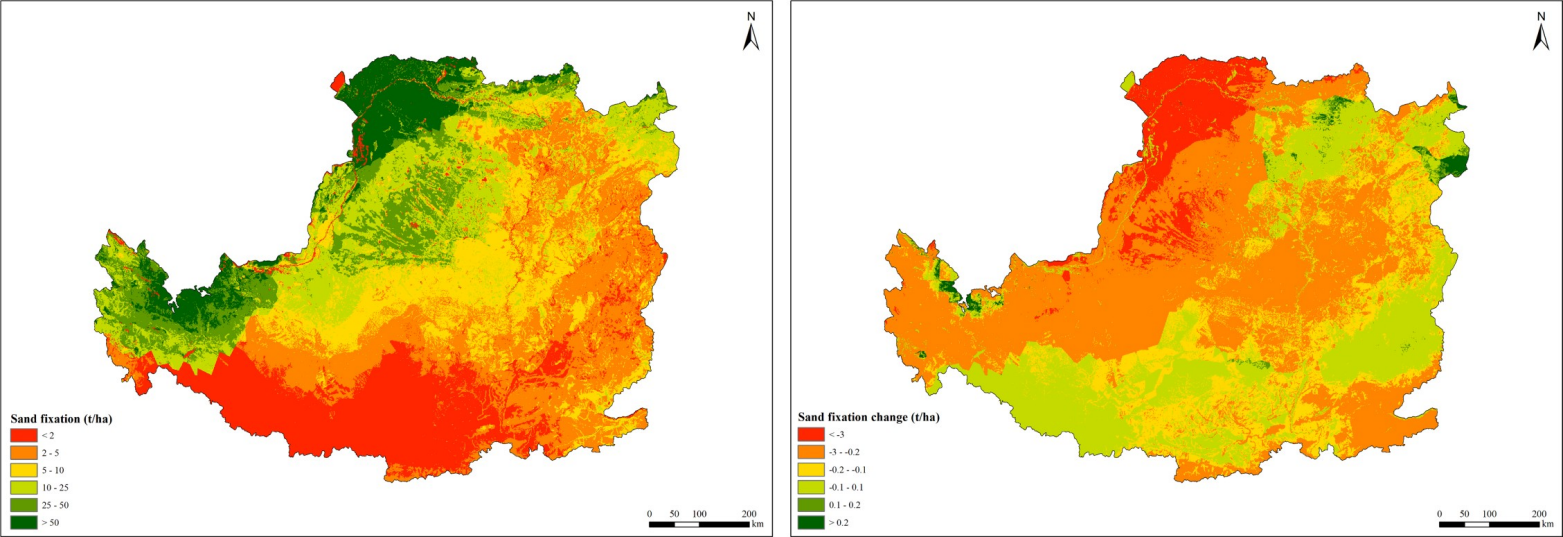
Spatial distribution of sand fixation in the Loess Plateau from 2000 to 2015.

From 2000 to 2015, the annual average sand fixation amount was 1.03 billion t/yr. Over this period, the per unit area of sand fixation was about 16.55 t/ha/yr, with a decreasing trend of 0.98 t/ha/yr. Sand fixation capacity of grassland ecosystems was 19.55 t/ha/yr, which was 1.83 times higher than that of forest ecosystems. Sand fixation capacity decreased from northwest to southeast. It showed spatial heterogeneity and was mainly distributed in the Middle Gansu and Ordos plateau covered by sandy lands and deserts, where wind erosion frequently occurred (Fig 7). In general, the changing trend of sand fixation during the study period showed a decrease in the Loess Plateau.

## Discussion

### Factors driving the ecosystem services

#### Effects of climate variability

**Fig 8:**
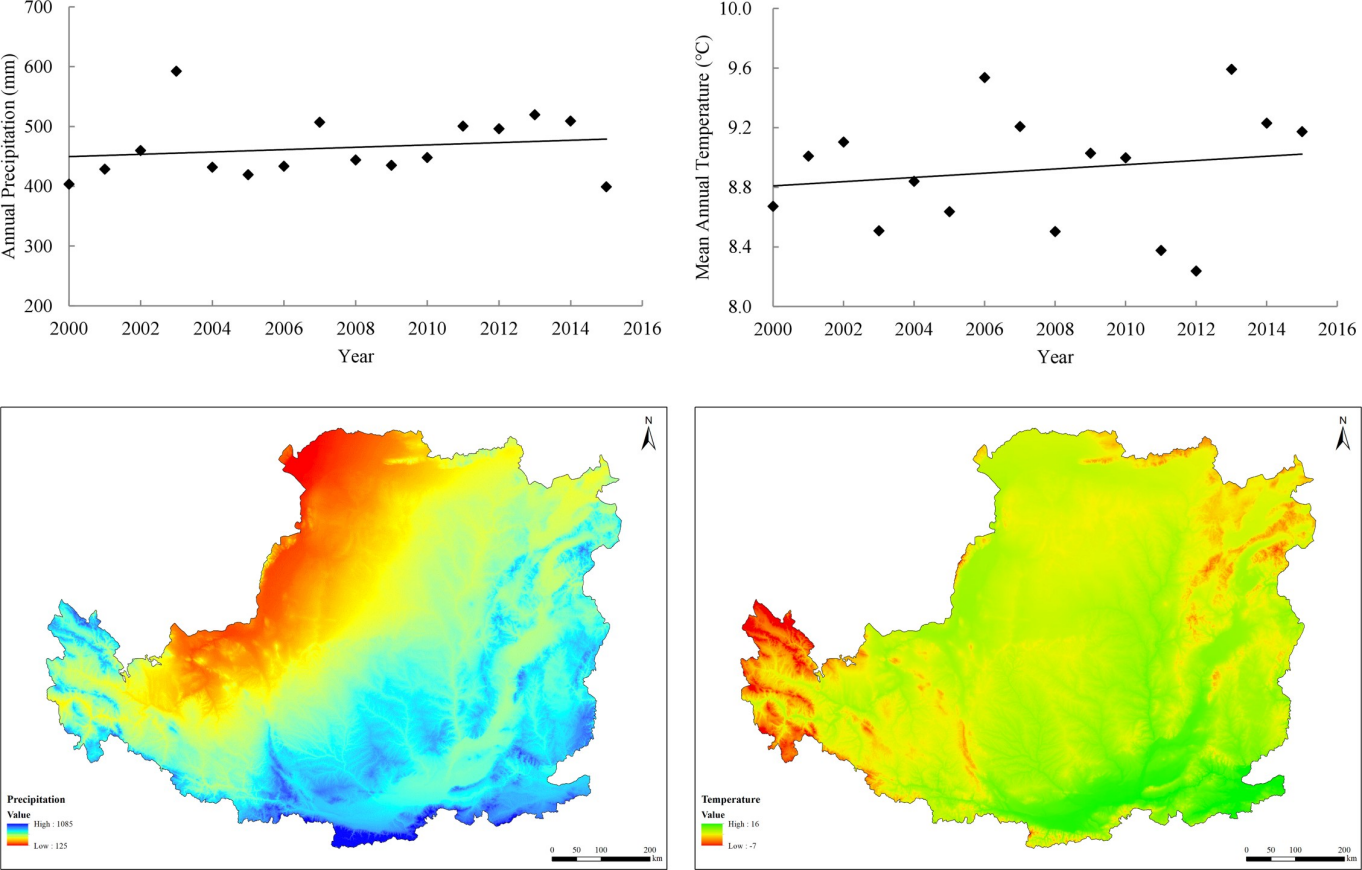
Temporal variations and spatial distributions of annual mean precipitation and temperature in the Loess Plateau from 2000 to 2015.

Climate and vegetation cover are direct and sensitive factors affecting hydrological processes, soil erosion, wind erosion and carbon sequestration [30–32]. Annual precipitation, temperature trends and spatial distributions from 2000 to 2015 in the Loess Plateau are presented in Fig 8. The annual mean precipitation and temperature was 464.27 mm and 8.92°C, respectively. Regional climate conditions in recent years exhibited a warming and wetting trend in the Loess Plateau. Precipitation was found to increase annually by an average of 1.96 mm and temperature was found to increase annually by an average of 0.01°C.

The warmer and wetter climate during 2000–2015 in the Loess Plateau improved vegetation growth and productivity. Ecosystem services such as soil conservation increased from northwest to southeast, corresponding with gradients in climate variability. Precipitation intensity and distribution was the main factor that most directly affecting water regulation. Regarding the parameters in the RUSLE and RWEQ models, the soil characteristics and topographic factors were relatively stable. So, the rainfall erosivity (R factor) and climate factors (WF factor) exhibited considerable variations. The annual mean R factor in the Loess Plateau increased at the rate of 26.65 MJ·mm/(t/ha/yr) from 2000 to 2015, which would theoretically cause more soil erosion. However, the soil conservation capacity had improved in the majority of the study area, which must be the result of increased forest land and grassland, in addition to vegetation restoration. Over this period, the annual mean sand fixation capacity decreased, which was mainly the result of the decrease in the WF factor, at a rate of 0.46 kg/m.

#### Effects of GTGP implementation

**Fig 9:**
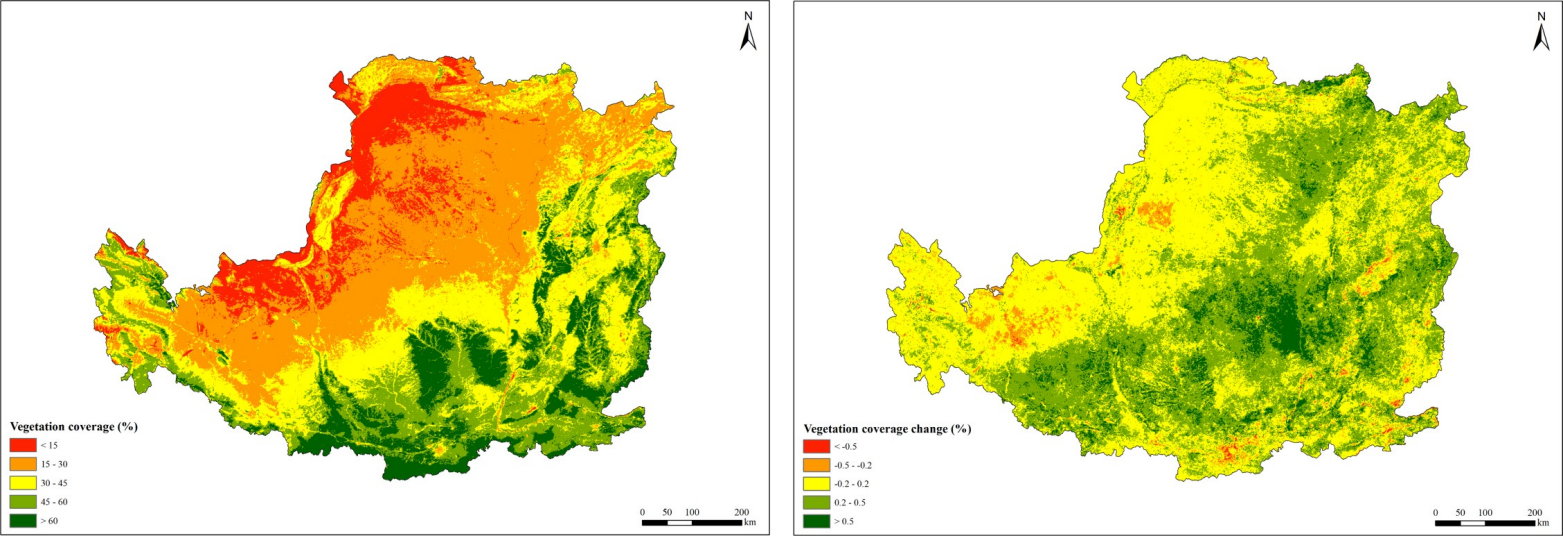
Spatial distribution of fractional vegetation cover (VC) in the Loess Plateau from 2000 to 2015.

The effects of GTGP implementation on ecosystem service in the Loess Plateau were mainly reflected by two aspects. First, it changed the ground cover directly through human activity. Human activity was now accepted as the main factor responsible for the transformation of the Earth’s surface [33–34]. Returning farmland to forest land and grassland, and expansion of construction land were the major causes of land use changes in the Loess Plateau, which could indirectly influence ecosystem services. Farmland area decreased by 10.58% during the study period. Studies have shown that the GTGP has fundamentally improved ecosystem services by increasing vegetation cover, decreasing water surface runoff and soil erosion, and reducing river sediments and nutrient loss to maintain soil fertility [35–37]. Quantitative analysis indicated that the rate of change of construction land increased up to 3.65%/yr, with expansion mainly from grassland or cropland. With the rapid increase in population growth and economic development, more extensive and intensive urban build-up, rural settlement and infrastructure construction would occupy ecological land, which would further reduce ecosystem services. Second, the implementation of GTGP changed regional VC in the Loess Plateau. VC can be used as a comprehensive quantitative indicator to show the growth status of vegetation brought about both by climate change and human activities. It is also an important parameter to assess the effects of ecological programs [38]. The spatial and slope distribution of annual mean VC data from 2000 to 2015 in the Loess Plateau is presented in Fig 9. Annual mean VC decreased from southeast to northwest. During the study period, vegetation cover restoration trends (slight + obvious restoration) accounted for approximately 45.85% of the Loess Plateau area, whereas the vegetation cover showing a decreasing trend (slight + obvious degradation) accounted for only 2.98% of the area; the remaining 51.17% of the area remained stable. These changes indicate that vegetation recovery might be achieved by the combined effects of wet-warmer climate change and the implementation of GTGP. The canopy could reduce the effect of rainfall on soil erosion, by its interception, and curtail the impact energy of raindrops to splash soil particles [39]. The litter layer affects surface runoff generation and soil water storage [40]. Soil permeability and strength under vegetation cover is considerably higher than that of bare soil [41], which could reduce soil erosion and wind erosion. To address the impact of vegetation recovery on ecosystem services in the Loess Plateau, average meteorological conditions from 2000 to 2015 and land use in 2015 were used to drive the models. The effects of vegetation restoration on water regulation, soil conservation and sand fixation was approximately 62.05%, 168.66% and 86.73%, respectively.

### Tradeoff and synergies between ecosystem services

Ecosystem management that attempts to maximize the production of one ecosystem service often results in substantial declines in the provision of other ecosystem services [42]. Thus, to better understand and take advantage of the relationship between ecosystem services, tradeoff and synergies need to be considered, from which human beings can benefit.

The correlation coefficient of the pairwise interactions between ecosystem services was shown in Table 1. VC has expanded because of a significant increase in grassland and forest land areas in the Loess Plateau. Both carbon sequestration and soil conservation showed a significant increase for most of the Loess Plateau area. The correlated changes indicate that they acted in synergy. The implementation of GTGP in the Loess Plateau might have contributed to the decrease in stream flow because of the potential increase in vegetation water consumption [43–44]. Thus, the decrease in regional water yield and the improvement in vegetation cover might be considered a tradeoff. Soil erosion mainly includes both wind and water erosion, so soil retention capacity is reflected mainly in the soil conservation and sand fixation. We found that regions with higher sand fixation modulus had lower soil conservation capacity (Figs 6 and 7). Consequently, the improvement in soil conservation and the decrease in regional sand fixation might be also a tradeoff.

**Table 1:** The correlation coefficient of the pairwise interactions between ecosystem services in the Loess Plateau.

	Carbon sequestration	Water regulation	Soil conservation	Sand fixation
Carbon sequestration	1.000	0.598*	0.577*	-0.763**
Water regulation		1.000	0.813**	-0.433
Soil conservation			1.000	-0.403
Sand fixation				1.000

Notes

*Correlation is significant at the 0.05 level (2-tailed).

**Correlation is significant at the 0.01 level (2-tailed).

### Uncertainties or limitations of this analysis

As the connections between ecosystem processes, functions and human wellbeing were complex and the various pathways were still not well understood, the simulation accuracy of ecosystem service was affected [7]. Firstly, the complex terrain of the Loess Plateau presented a challenge for deriving spatial distribution of annual climate factors that were interpolated from records of 136 meteorological stations at a 1-km resolution. Secondly, seasonal and inter-annual variability of meteorological factors, water resource used by communities, impacts of soil conservation structures were not considered in this paper [8]. Thirdly, the soil conservation estimation was undertaken through the RUSLE, which was based on a statistical relationship established from many plot scale rainfall-erosion experiments. This effect may have been overestimated in this research due to the omission of the local sediment deposition process [45]. Finally, the sand fixation assessment was undertaken through the RWEQ, which was an empirical-based model to estimate wind erosion at field scale in the USA. The classification of soil particle-size between the United States and China, topographic factors or determination of the erodibility boundary need to be further researched [46]. To improve the simulation accuracy of ecosystem services, provincial meteorological station data of the Loess Plateau, higher spatial and temporal resolutions data and localized parameters would be useful in future research.

### Sustainability through adaptive management

One of the aims of the GTGP was to reduce the burden on land that might have been cultivated. Since 2000, considerable labor, material and financial resources have been put into vegetation rehabilitation in the Loess Plateau. Our measurements of vegetation coverage and ecosystem services indicate that these inputs have been effective in halting further ecological degradation of this region and a change in the direction of recovery. However, as the sustainability of ecological rehabilitation efforts depend on scientific understanding of interactions between people and their surrounding ecosystems [47], there are still some problems and concerns in the implementation of the programs.

Compensation by allocation of grain and cash subsidies to households that have to stop cultivation of farmland and use of wood from forests is an effective practice to implement these programs. This encourages people who depend on production from the land for their livelihood to take part in ecological protection and construction. However, annual subsidies have mostly remained constant while prices of products and labor have continued to increase. Thus, it is very important to develop alternative industries to increase the incomes of the numerous farmers of the region to solve their long-term livelihood problems [48].

Estimation the correlations showed trade-offs and synergy among different kinds of ecosystem services, which is crucial for ecosystem services management. Grain for green and vegetation recovery had remarkable effects on ecosystem services. However, to balance green and grain trade, and water supply and consumption, vegetation in the Loess Plateau should be maintained but not expanded further as planned [49]. The strategy for the future needs to emphasize protection and better management of existing vegetation and natural succession. As water limitation is an important factor in restricting vegetation restoration in the Loess Plateau, returning farmland to forest land or grassland should consider the site conditions and water characteristics. Highly water-consuming exotic species should gradually be replaced by indigenous species.

## Conclusions

This paper assessed the effects of ecological restoration in the Loess Plateau through spatial and temporal changes in land use and ecosystem services (carbon sequestration, water regulation, soil conservation and sand fixation) from 2000 to 2015. The main conclusions are as follows. (1) As a result of the GTGP, the area of forest land and grassland significantly expanded, while the area of farmland substantially decreased. (2) Ecosystem services showed overall improvement with localized deterioration, because of wetter and warmer climate variability in recent years and the positive effects of ecological restoration. (3) There were synergies between carbon sequestration and water regulation, and tradeoff between soil conservation and sand fixation. Therefore, better understanding the relationships among ecosystem services could promote the sustainable use of ecosystem services and facilitate policy-making.

Overall, the GTGP implemented in the Loess Plateau has substantially contributed to vegetation recovery. The increased forest land and grassland area and vegetation coverage in recent years have promoted ecosystem services. However, vegetation and water resources typically maintain an inverse relationship in semi-arid water-limited environments. Water shortage issues should be given high priority. Also, long-term management to encourage sustainable development is necessary to maintain and consolidate the positive effects of the ecological programs.

## Supporting information

S1 Fig Spatial distribution of parameters used for water regulation assessment. (a) Rainfall, (b) the percentage of annual output of flow rainfall to precipitation amounts, and (c) the profit coefficient of ecosystems’ decreasing runoff.
(TIF)

S2 Fig Spatial distribution of parameters used for soil conservation assessment. (a) Rainfall erodibility, (b) soil erodibility, (c) the LS-slope-length factor, (d) vegetation cover factor, and (e) erosion control practice factor.
(TIF)

S3 Fig Spatial distribution of parameters used for sand fixation assessment. (a) Climate factor, (b) soil erodibility factor, (c) soil crust factor, (d) soil roughness factor, and (e) vegetation cover factor.
(TIF)
