# Supplementary figures and images for: Ecosystem services changes between 2000 and 2015 in the Loess Plateau, China: A response to ecological restoration
Source: PLoS One. 2019 Jan 28;14(1):e0209483. doi: 10.1371/journal.pone.0209483 (PMC6349322; doi:10.1371/journal.pone.0209483)

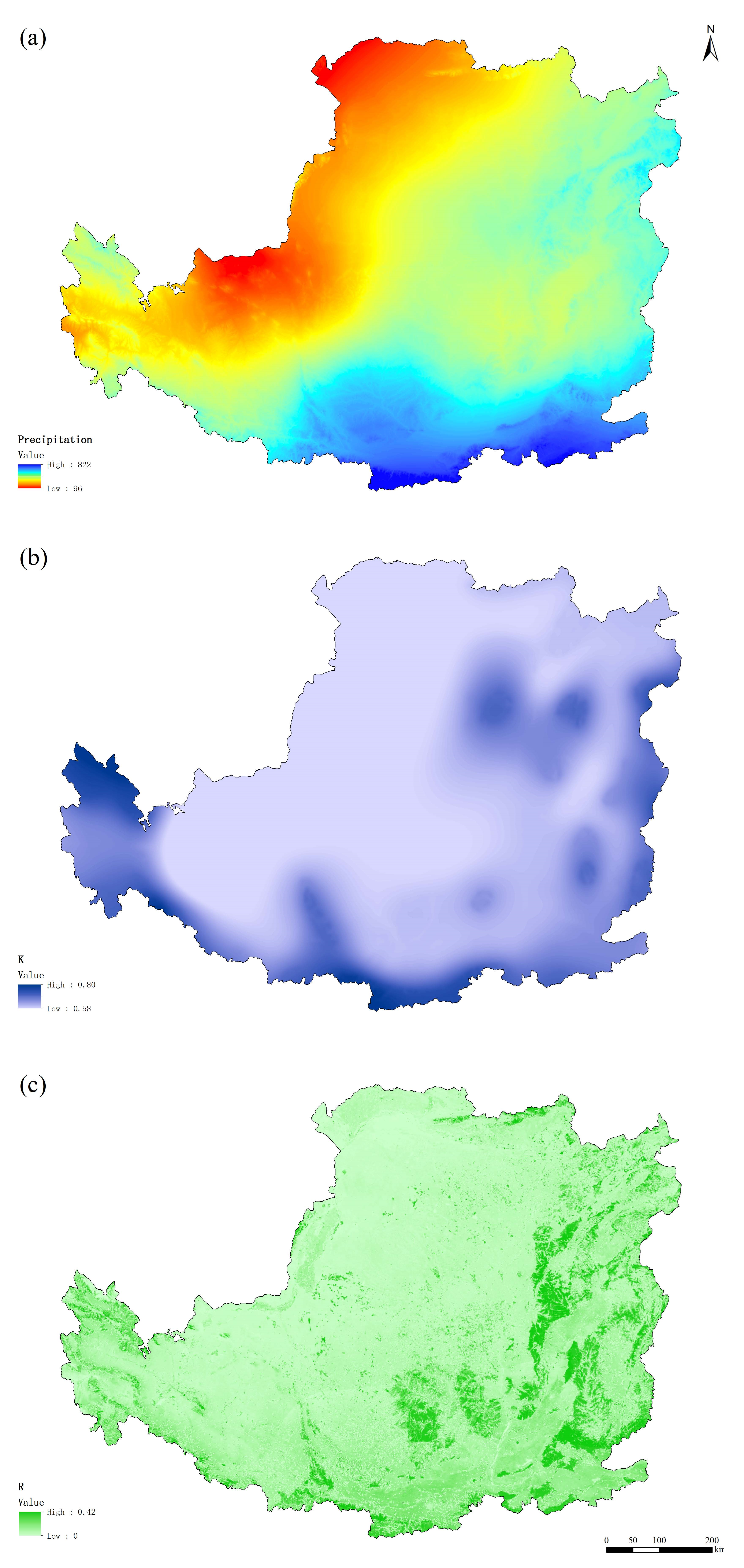

Supplement: S1 Fig — (a) Rainfall, (b) the percentage of annual output of flow rainfall to precipitation amounts, and (c) the profit coefficient of ecosystems’ decreasing runoff. (TIF) [file pone.0209483.s001.tif]

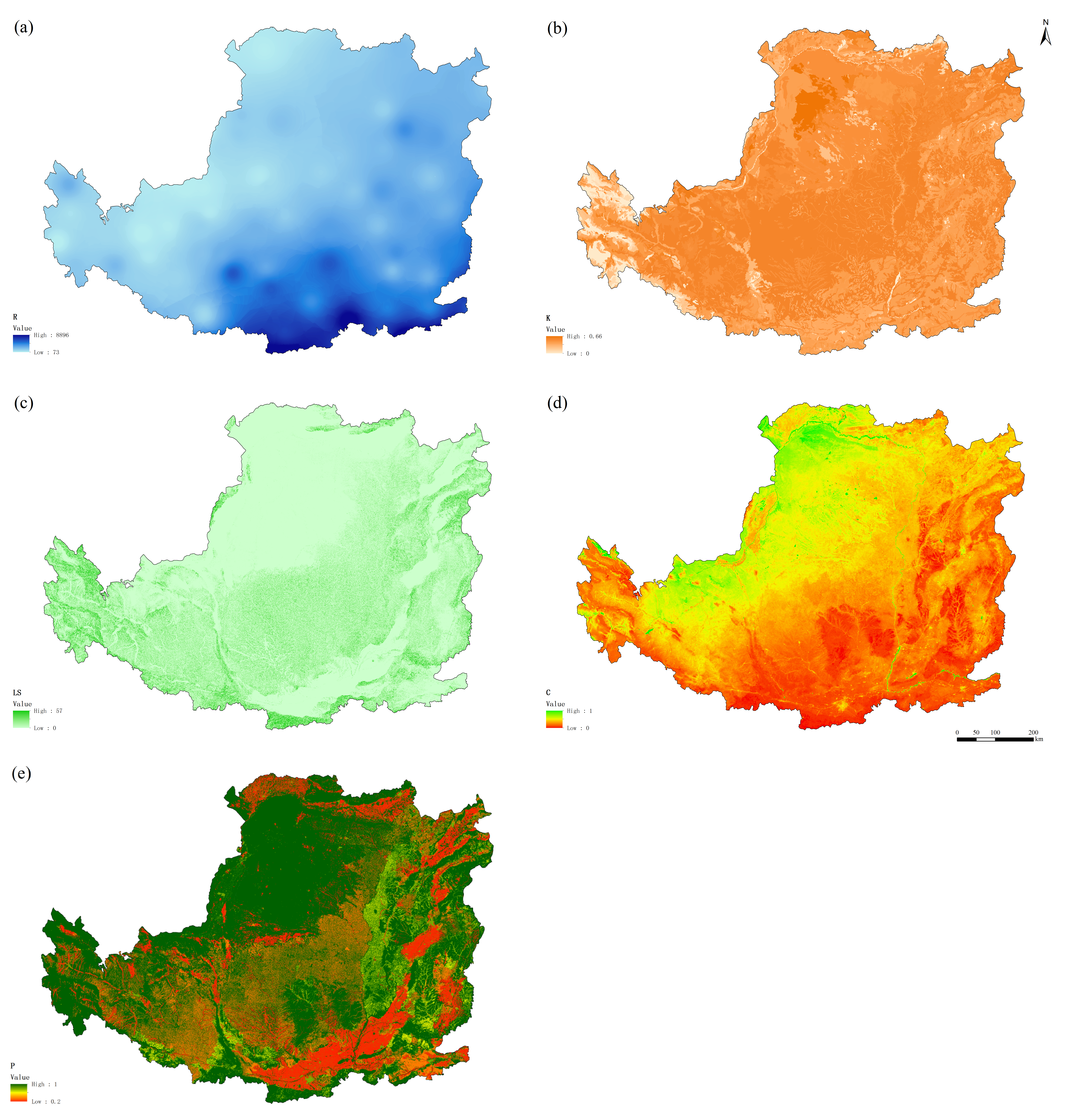

Supplement: S2 Fig — (a) Rainfall erodibility, (b) soil erodibility, (c) the LS-slope-length factor, (d) vegetation cover factor, and (e) erosion control practice factor. (TIF) [file pone.0209483.s002.tif]

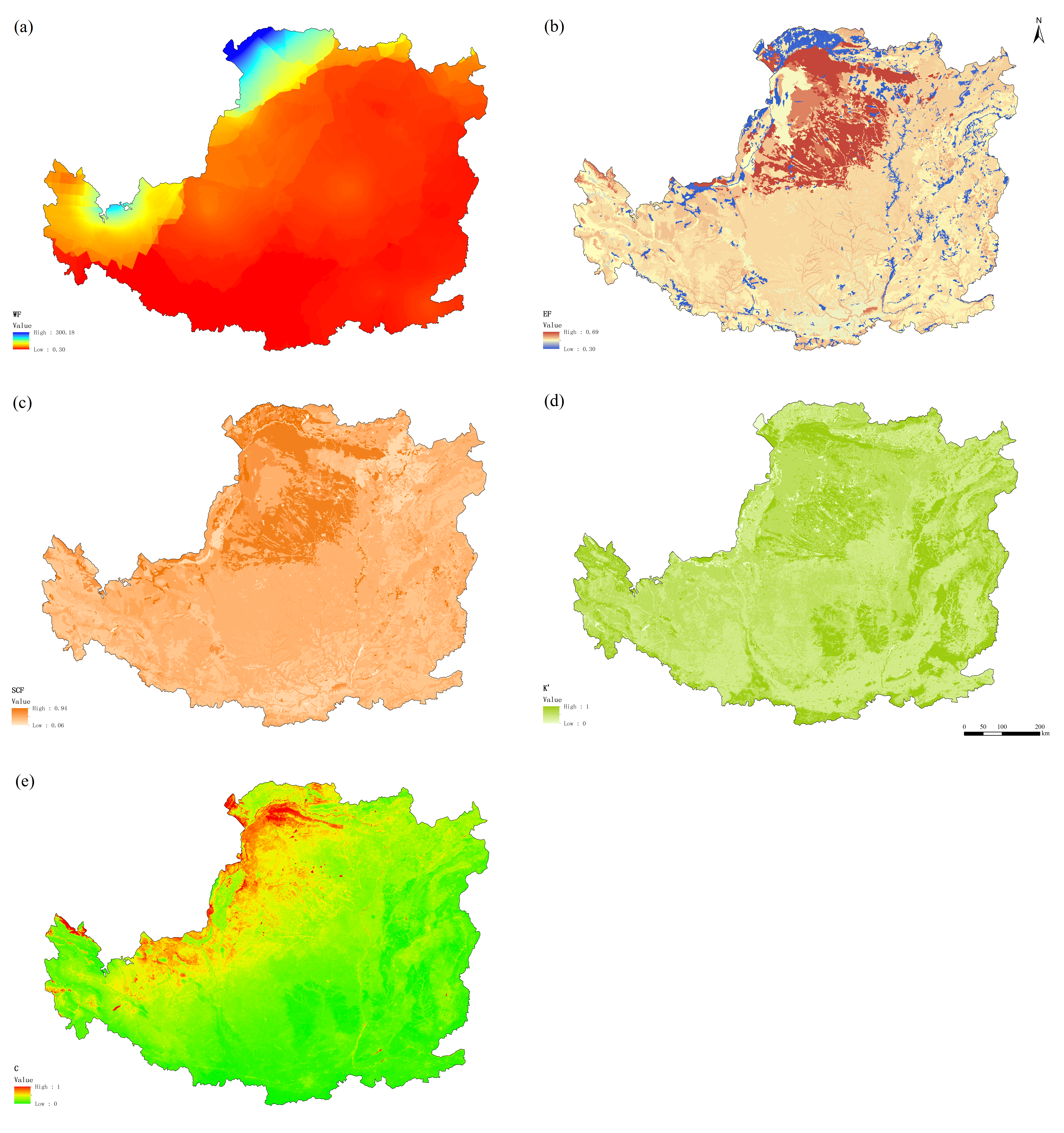

Supplement: S3 Fig — (a) Climate factor, (b) soil erodibility factor, (c) soil crust factor, (d) soil roughness factor, and (e) vegetation cover factor. (TIF) [file pone.0209483.s003.tif]
